# Supplementary material for: Association of Handgrip Strength and Nutritional Status in Non-Dialysis-Dependent Chronic Kidney Disease Patients: Results from the KNOW-CKD Study
Source: Nutrients. 2024 Jul 26;16(15):2442. doi: 10.3390/nu16152442 (PMC11314453; doi:10.3390/nu16152442)

Supplementary Materials

Association of Handgrip Strength and Nutritional Status in Non-Dialysis-Dependent Chronic Kidney Disease Patients: Results from the KNOW-CKD Study

M Kim et al.

Table of contents

Method S1. Anthropometric measurements included in nutritional assessment

Table S1. Multivariate correlation analysis between handgrip strength and other nutritional parameters.

Table S2. Sensitivity analysis results of the association between handgrip strength and malnutrition-inflammation score

Figure S1. Predictability of the maximum and average value of handgrip strength for diagnosing malnutrition in men and women.

Method S1

Anthropometric measurements included in nutritional assessment

Skinfold thickness (SFT) was measured from four areas: biceps, triceps, subscapular, and suprailiac, preferably measured directly on bare skin using a caliper (Skyndex Digital Caliper). The biceps and triceps SFT were measured with the patient standing upright and palm facing forward, at the midpoint between the tip of the shoulder and the tip of the elbow. The subscapular SFT was measured approximately 2–3cm below the most prominent part of the scapula on the patient's back. The suprailiac SFT was measured approximately 2–3cm above the most prominent part of the pelvic bone.

Mid-arm circumference (MAC) was measured with the patient wearing as thin clothing as possible and assuming upright position. A tape measure was wrapped around at the midpoint between the tip of the shoulder and the tip of the elbow to measure the MAC. Moreover, mid-arm muscle circumference (MAMC) was calculated using the following formula: MAMC = MAC – (3.14 × Triceps SFT).

Table S1. Multivariate correlation analysis between handgrip strength and other nutritional parameters.

	ρ (95% CI)	P-value	R	R^2
**BMI**	0.27 (0.18, 0.36)	<0.001	0.27	0.07
**SFT (Biceps)**	-0.17 (-0.26, -0.07)	0.001	-0.17	0.03
**SFT (Triceps)**	-0.25 (-0.34, -0.16)	<0.001	-0.25	0.06
**SFT (Subscapular)**	0.08 (-0.02, 0.18)	0.11	0.08	0.01
**SFT (Suprailiac)**	-0.01 (-0.11, 0.09)	0.82	-0.01	<0.01
**MAMC**	0.60 (0.53, 0.66)	<0.001	0.60	0.36
**Intracellular water**	0.78 (0.74, 0.81)	<0.001	0.78	0.60
**Extracellular water**	0.71 (0.66, 0.76)	<0.001	0.71	0.51
**Total body water**	0.76 (0.71, 0.80)	<0.001	0.76	0.57
**Fat mass**	0.01 (-0.09, 0.10)	0.93	<0.01	<0.01
**Fat-free mass**	0.76 (0.71, 0.80)	<0.001	0.76	0.57
**Soft lean mass**	0.76 (0.72, 0.80)	<0.001	0.76	0.58
**Serum albumin**	0.07 (-0.03, 0.17)	0.15	0.07	0.01
**Hemoglobin**	0.46 (0.38, 0.53)	<0.001	0.46	0.21

CI, confidence interval; BMI, body mass index; SFT, skinfold thickness; MAMC, mid-arm muscle circumference.

Table S2. Sensitivity analysis results of the association between handgrip strength and malnutrition-inflammation score

	Unadjusted model		Multivariable model 1		Multivariable model 2		Multivariable model 2 with imputation[1]	
**HGSmax**								
	**β (95% CI)**	**P**	**β (95% CI)**	**P**	**β (95% CI)**	**P**	**β (95% CI)**	**P**
MIS (as continuous variable)	-0.32 (-0.49, -0.15)	<0.001	-0.43 (-0.70, -0.16)	0.002	-0.46 (-0.77, -0.14)	0.005	-0.43 (-0.70, -0.16)	0.002
	**OR (95% CI)**	**P**	**OR (95% CI)**	**P**	**OR (95% CI)**	**P**	**OR (95% CI)**	**P**
MIS ≥ 6	0.47 (0.30, 0.75)	0.002	0.33 (0.16, 0.69)	0.003	0.41 (0.19, 0.89)	0.025	0.35 (0.16, 0.74)	0.007
MIS ≥ 5	0.60 (0.43, 0.86)	0.005	0.54 (0.31, 0.93)	0.025	0.57 (0.32, 1.03)	0.06	0.53 (0.30, 0.93)	0.028
**HGSavg**								
	**β (95% CI)**	**P**	**β (95% CI)**	**P**	**β (95% CI)**	**P**	**β (95% CI)**	**P**
MIS (as continuous variable)	-0.32 (-0.49, -0.14)	<0.001	-0.38 (-0.65, -0.12)	0.004	-0.40 (-0.71, -0.10)	0.010	-0.39 (-0.65, -0.12)	0.005
	**OR (95% CI)**	**P**	**OR (95% CI)**	**P**	**OR (95% CI)**	**P**	**OR (95% CI)**	**P**
MIS ≥ 6	0.50 (0.31, 0.79)	0.003	0.40 (0.20, 0.81)	0.010	0.48 (0.23, 1.01)	0.05	0.42 (0.21, 0.86)	0.018
MIS ≥ 5	0.60 (0.42, 0.86)	0.005	0.56 (0.33, 0.95)	0.032	0.58 (0.33, 1.03)	0.06	0.55 (0.32, 0.95)	0.032

HGSmax is the maximum value and HGSavg is the average value of handgrip strength measured twice on each hand. The odds ratios (OR) and beta coefficients are standardized with 1 standard deviation increase of HGSmax or HGSavg. Multivariable model 1 was adjusted for age, sex, history of diabetes mellitus and hypertension, stage of chronic kidney disease, smoking status, and overhydration. Multivariable model 2 was adjusted for age, sex, history of diabetes mellitus and hypertension, stage of chronic kidney disease, smoking status, overhydration, income status, and education status. HGS, handgrip strength; MIS, malnutrition-inflammation score; CI, confidence interval.
1. The multivariate imputation by chained equations method was used to impute the covariate of income status.

Figure S1. Predictability of the maximum and average value of handgrip strength for diagnosing malnutrition in men and women.

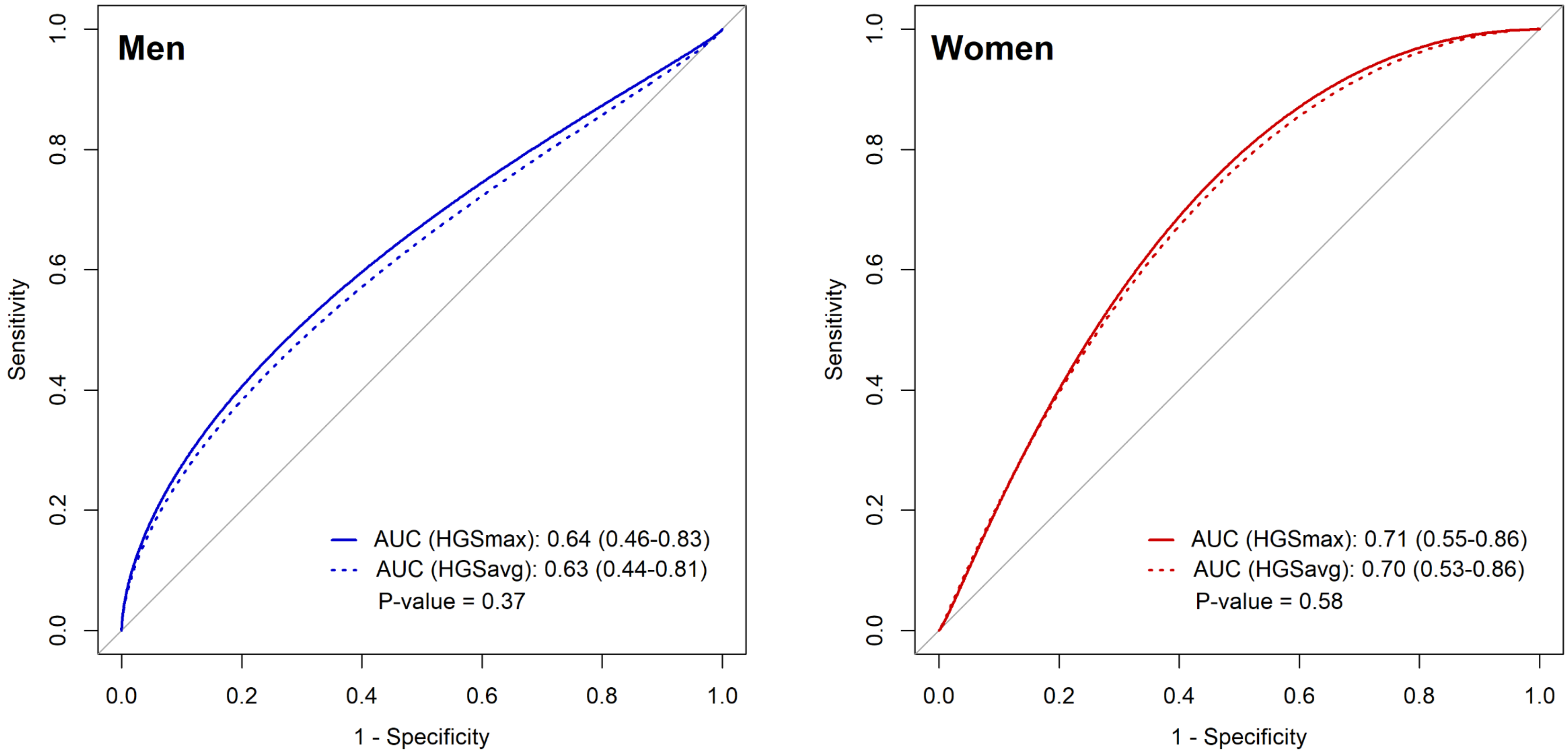

Supplement: Supplementary file 1 [file nutrients-16-02442-s001.zip › nutrients-3093975-supplementary.pdf]
